# Electron–Phonon Coupling and Nonthermal Effects in Gold Nano-Objects at High Electronic Temperatures

**DOI:** 10.3390/ma15144883

**Published:** 2022-07-13

**Authors:** Nikita Medvedev, Igor Milov

**Affiliations:** 1 Institute of Physics, Czech Academy of Sciences, Na Slovance 1999/2, 18221 Prague, Czech Republic; 2 Institute of Plasma Physics, Czech Academy of Sciences, Za Slovankou 3, 18200 Prague, Czech Republic; 3 Advanced Research Center for Nanolithography (ARCNL), Science Park 106, 1098 XG Amsterdam, The Netherlands; i.milov@arcnl.nl; 4 Center for Free-Electron Laser Science CFEL, Deutsches Elektronen-Synchrotron DESY, 22607 Hamburg, Germany

**Keywords:** electron–phonon coupling, nanoparticle, ultrathin layer, nonthermal melting, tight-binding molecular dynamics, Boltzmann collision integrals, XTANT

## Abstract

Laser irradiation of metals is widely used in research and applications. In this work, we study how the material geometry affects electron–phonon coupling in nano-sized gold samples: an ultrathin layer, nano-rod, and two types of gold nanoparticles (cubic and octahedral). We use the combined tight-binding molecular dynamics Boltzmann collision integral method implemented within XTANT-3 code to evaluate the coupling parameter in irradiation targets at high electronic temperatures (up to *T_e_*~20,000 K). Our results show that the electron–phonon coupling in all objects with the same fcc atomic structure (bulk, layer, rod, cubic and octahedral nanoparticles) is nearly identical at electronic temperatures above *T_e_*~7000 K, independently of geometry and dimensionality. At low electronic temperatures, reducing dimensionality reduces the coupling parameter. Additionally, nano-objects under ultrafast energy deposition experience nonthermal damage due to expansion caused by electronic pressure, in contrast to bulk metal. Nano-object ultrafast expansion leads to the ablation/emission of atoms and disorders the inside of the remaining parts. These nonthermal atomic expansion and melting are significantly faster than electron–phonon coupling, forming a dominant effect in nano-sized gold.

## 1. Introduction

Manipulating material properties and creating materials and devices with new functionality on the nanoscale is a driving force in modern nanotechnology [1]. Using a strong external stimulus such as an intense laser pulse, it is possible to induce exotic transient states of matter that upon relaxation result in materials with altered optical, thermal, mechanical, or chemical properties. Ultrashort laser pulses are used in materials nanostructuring for applications in plasmonics [2], catalysis [3], photovoltaics [4], and biomedicine [5]. Reversible laser-induced switching of material structure between crystalline and amorphous phases can be used for efficient data storage [6]. Achieving precise control of the outcome of laser-matter interaction is one of the ultimate goals in the ultrafast laser community [7]. Such control is not possible without a deep understanding of the fundamental processes involved.

When an intense ultrashort laser pulse is absorbed by a solid, it results in a strong excitation of the electronic system, which, upon fast thermalization, can be described as an increased electronic temperature with respect to the lattice temperature [8,9]. Such hot electrons exchange thermal energy with the lattice via the process known as electron–phonon coupling [8,10]. Additionally, strongly excited electrons can directly influence the atomic potential energy surface, resulting in nonthermal atomic movements [11,12,13,14]. Thermal and nonthermal energy exchange between electrons and atoms is intertwined and may occur on a similar timescale in laser-irradiated materials, which makes it challenging to study theoretically [15,16] and observe separately in experiments [12,17,18].

Several recent studies showed that nonthermal phase transitions in metals are possible when the expansion of irradiated material is allowed [14,19,20]. Such expansion caused by electron pressure destabilizes the lattice and may be observed in small nano-objects where expansion is significant. However, the expansion must be faster than the electron–phonon energy exchange rate, so that nonthermal effects outrun the conventional thermal melting.

Apart from the fundamental interest in studying the interplay between thermal and nonthermal effects in laser-irradiated metallic nano-objects, reducing the size of functional devices is a dominant trend in nanotechnology applications. Such downscaling, however, contains many challenges in the fabrication and understanding of nano-materials behavior under extreme laser-induced conditions, which can be considerably different than their bulk counterparts. A bright example is the ability of metallic nanoparticles to amplify, concentrate and manipulate light due to the excitation of surface plasmon resonances [21,22].

In this theoretical work, we investigate the role of a nano-object shape and dimensionality in its ultrafast laser-induced thermal and nonthermal dynamics in the example of gold. We calculate the electron–phonon coupling parameter and electron heat capacity as functions of the electron temperature and demonstrate scenarios of nonthermal damage in gold nanolayer, nanorod, and cubic and octahedral nanoparticles.

## 2. Materials and Methods

To trace the atomic response of gold to excitation of the electronic system (an increase in the electronic temperature), we use a hybrid code XTANT-3 [23]. The code combines the following models in a unified approach:

(a) A transport Monte Carlo (MC) model calculating photoabsorption and nonequilibrium kinetics of high-energy electrons. This module only serves to deliver energy into the electronic system within the current work. We will not be focusing on the nonequilibrium electron cascades stage, and we assume electrons to be always in local equilibrium.
(b) Rate equations tracing the distribution function of the low-energy electron fraction (electron populations on the transient band structure). The rate equations include source terms of energy delivery from the MC particles [24], and electron–ion (electron–phonon) energy exchange [10].
(c) Boltzmann collision integrals calculating the coupling between the low-energy electrons and the atomic motion (electron–phonon coupling) [10]. It forms a source term due to the energy exchange in the rate equations.
(d) A transferable tight-binding (TB) method calculating the transient band structure of the material and the inter-atomic potential. We apply the Naval Research Laboratory (NRL) tight-binding parameterization to model gold, which uses an sp^3^d^5^ linear combination of atomic orbitals (LCAO) basis [25,26].
(e) Classical molecular dynamics (MD) simulation tracing atomic motion [23]. The potential for atoms is obtained from the TB method, accounting for the transient electronic populations calculated with the rate equations. The energy from electrons (electron–phonon coupling) is transferred to atoms via velocity scaling at each time step of the simulation [10].

Neglecting the nonequilibrium electronic cascades stage, we assume that the low-energy fraction of electrons follows the Fermi–Dirac distribution, $f\left(E\right)=2{\left(1+\exp\left(\left(E-\mu \left(T_{e}\right)\right)/k_{B}T_{e}\right)\right)}^{-1}$, at all times (here μ(*T_e_*) is the chemical potential of electrons, *T_e_* is the electronic temperature, $k_{B}$ is the Boltzmann constant, and the factor of 2 accounts for the spin degeneracy). To calculate the electron–phonon coupling parameter, we use the methodology developed in [10]. The coupling parameter is defined as:

(1) $$G\left(T_{e},T_{a}\right)=\frac{1}{V\left(T_{e}-T_{a}\right)}\underset{i,j}{\sum }E_{i}I_{e-a}^{ij},$$

where *T_a_* is the atomic temperature, *E_i_* is the electronic energy levels (eigenstates of the transient Hamiltonian, $E_{i}=\langle i|H|i\rangle$), *V* is the volume of the modeled sample, and the Boltzmann collision integral $I_{e-a}^{ij}$ takes the following form for the coupling of degenerate electrons to classical atoms [10]:

(2) $$I_{e-a}^{ij}=w_{ij}\{\begin{array}{c} f\left(E_{j}\right)\left(2-f\left(E_{i}\right)\right)e^{-E_{ij}/T_{a}}-f\left(E_{i}\right)\left(2-f\left(E_{j}\right)\right),\;\mathrm{for}\;i>j\\ f\left(E_{j}\right)\left(2-f\left(E_{i}\right)\right)-f\left(E_{i}\right)\left(2-f\left(E_{j}\right)\right)e^{-E_{ji}/T_{a}},\;\mathrm{for}\;i<j. \end{array}$$

Here, *w_ij_* is the rate of electron transitions triggered by an arbitrary atomic displacement; *E_ij_* = *E_i_* − *E_j_* is the difference between the energies of the two electronic levels participating in an electron transition; and $f\left(E_{i}\right)$ is the electronic distribution function (electron population on the energy level *E_i_*). The electron transition rates are defined by the sudden change of the Hamiltonian due to atomic displacement during a given time step [10]:

(3) $$w_{ij}=\frac{4e}{\hbar \delta t^{2}}\underset{\alpha ,\beta }{\sum }{\left|c_{i,\alpha }\left(t\right)c_{j,\beta }\left(t_{0}\right)S_{\alpha ,\beta }\right|}^{2},\;$$

where *e* is the electron charge, *ħ* is the Planck’s constant, $c_{i,\alpha }$ are the coefficients of the expansion of the electronic wave function $\psi_{i}$ of the state *i* in the linear combination of the atomic orbitals (LCAO) basis set within the TB model: $\psi_{i}=\sum_{\alpha }c_{i,\alpha }\varphi_{\alpha }$ ($\varphi_{\alpha }$ are the basis set functions). The expansion coefficients are calculated at the consecutive MD time steps *t*_0_ and *t* = *t*_0_ + *δt*. $S_{\alpha ,\beta }$ is the TB overlap matrix, calculated at the same time step.

The used method does not imply phononic approximation. Instead, we calculate electronic transitions in response to any atomic displacement—anharmonic atomic motion, nonperiodic systems, and noncrystalline (including amorphous and liquid states) can be modeled with the same approach. Thus, even though we use the common term “electron–phonon” coupling, it is in fact a more general electron–ion coupling. For generality and simplifying further reading, to denote *G* from Equation (1) we use the term “coupling parameter” in the rest of the work.

The electronic heat capacity is calculated as a derivative of the electron entropy with respect to the electron temperature at a constant volume. For the Fermi–Dirac distribution, $f\left(E_{i}\right)$, it reduces to the standard expression:

(4) $$C_{e}\left(T_{e},T_{a}\right)=\frac{1}{V}\underset{i}{\sum }\frac{\partial f\left(E_{i}\right)}{\partial T_{e}}\left(E_{i}-\mu \left(T_{e}\right)\right),$$

where for the calculation of $\partial f\left(E_{i}\right)/\partial T_{e}$, the derivative of the electronic chemical potential by the electronic temperature $\partial \mu \left(T_{e}\right)/\partial T_{e}$ is calculated numerically [27].

We analyze a nano-layer of gold of a thickness of 1.62 nm, which consists of 4 × 4 × 4 orthogonal unit cells (256 atoms) with open surfaces along the Z-axis and periodic boundary conditions along X and Y. For a gold nano-rod with a square cross-section, we used the same setup, but with a periodic boundary condition along the X-axis only. For a cubic nanoparticle (NP), no periodic conditions were imposed.

As a more realistic setup, we also used the truncated octahedral gold NP enclosed by {100} and {110} facets [28]. A gold NP with a width of 1.62 nm (249 atoms) was simulated. The NP was constructed with the help of the NanoCrystal web-based tool [28].

**Figure 1:**
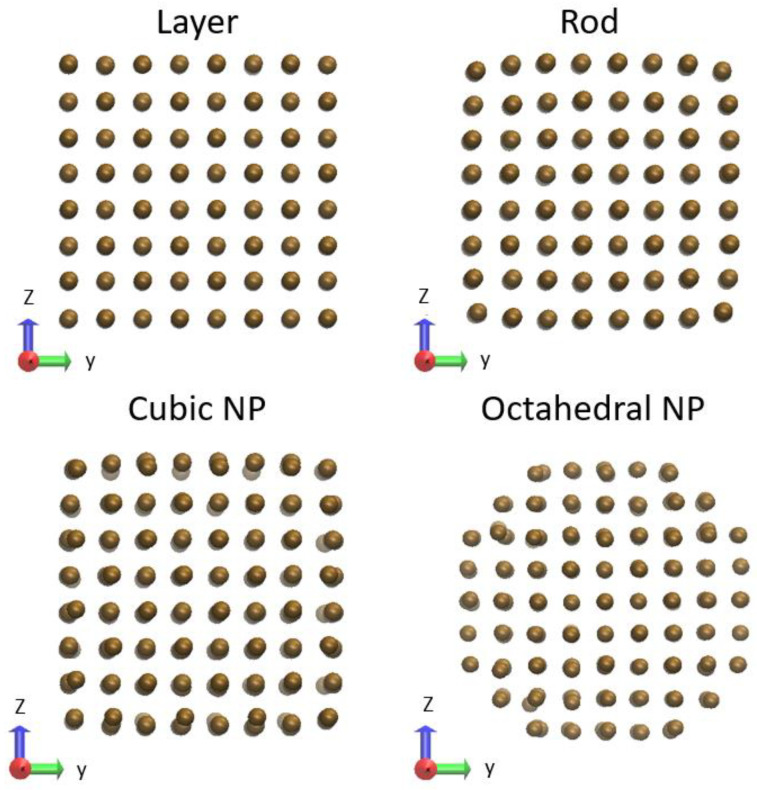
Snapshots of initial atomic positions used for simulation of gold: nano-layer (periodic boundary conditions in *x* and *y* directions), nano-rod (periodic boundary conditions in the *x* direction), cubic, and octahedral NPs (no periodic boundary conditions) as used in XTANT-3 simulation.

All the systems were relaxed with the zero-temperature MD (steepest descent algorithm) before productive simulation runs. The respective set-ups are shown in Figure 1. Starting from these atomic positions and Maxwellian velocity distributions, the systems are thermalized at room temperature for a few hundred femtoseconds before the increase in the electronic temperature used to extract the coupling parameter and the electron heat capacity (see Appendix A for more details). We use an NVE (microcanonical) ensemble. The simulation uses a time step of 0.2 fs. Atomic snapshots are visualized with the help of the VMD software [29].

Following the developed methodology [10], ten dynamical XTANT-3 simulation runs are performed with different initial conditions and parameters of electron temperature increase (various irradiation durations and aimed electronic temperatures). The presented coupling parameters and heat capacities are averaged over the ten simulation runs. The standard deviations of these ten runs define the error bars in the calculations.

## 3. Results

### 3.1. Thermal Parameters

**Figure 2:**
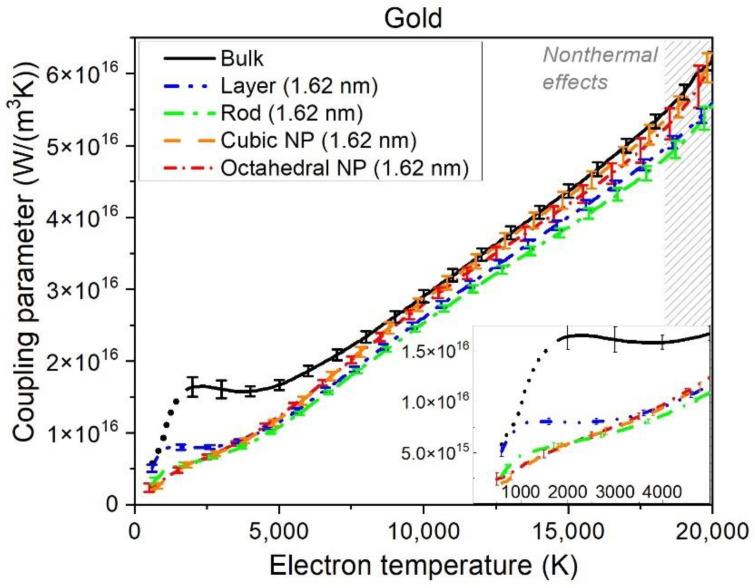
Electron–ion (electron–phonon) coupling parameter in gold bulk, layer (1.62 nm thick), rod with a cubic cross-section (1.62 × 1.62 nm^2^), cubic NP (1.62 nm), and octahedral NP (1.62 nm) as functions of the electronic temperature, calculated with XTANT-3. The dotted low-temperature part of the bulk curve indicates the region where calculations may not be reliable. The shaded area at high electronic temperatures shows a region where nonthermal effects become noticeable (see text). Error bars are standard deviations in the ten simulation runs. The inset zooms into the low-temperature region of the same figure.

Figure 2 shows calculated coupling parameters in gold NPs (octahedral and cubic), nano-rod, and nano-layer compared with bulk gold from [10]. All the coupling parameters practically coincide within the error bars at electronic temperatures above *T_e_*~7000 K, demonstrating nearly linear growth with *T_e_*. We conclude that at high electronic temperatures the coupling parameter is not sensitive to the sample sizes, dimensions, and geometries.

At lower electronic temperatures, two regions can be detected—fast linear growth and saturation plateau. The value and width of the plateau seem to increase with the increase in dimensionality (bulk > layer > rod > NP). The plateau also starts at lower *T_e_* for smaller dimensionality. Overall, at low *T_e_*, the difference between a layer, a rod, and NPs is minor, with only bulk gold being noticeably different.

One should note that, at *T_e_* < 2000 K, the calculations may be unreliable (marked with a dotted line for bulk gold). As discussed in [10], this is because, at low electronic temperatures, the edge of the Fermi–Dirac distribution function only overlays with a small number of the electronic energy levels, calculated at the Г-point of the Brillouin zone of the bulk. It may be insufficient to reliably calculate the coupling parameter. This method of calculation requires the electronic temperature to be sufficiently larger than the energy difference between the nearest electron energy levels around the Fermi energy: *T_e_* >> *E_i+1_*–*E_i_*. In practice, for the simulation box sizes we used, it means the electronic temperature of ~2000 K for bulk simulation. We return to that issue in the Discussion section.

**Figure 3:**
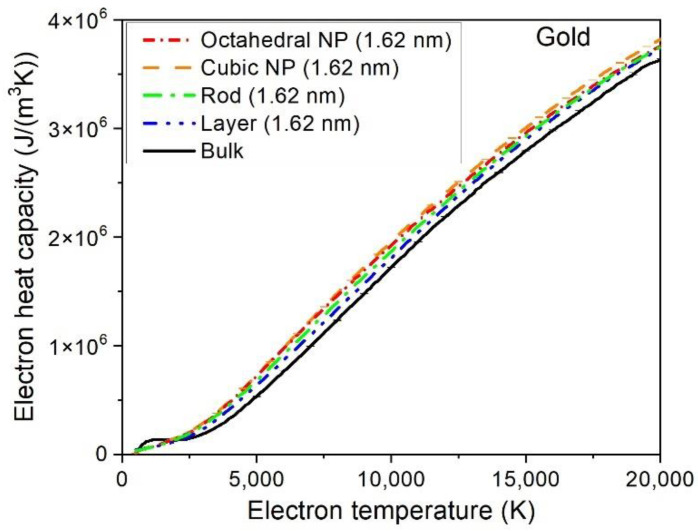
Electronic heat capacity in gold nanoparticles (NP, 1.62 nm width) and a thin layer (1.6 nm thick) vs. bulk gold as functions of the electronic temperature, calculated with XTANT-3. Error bars are of a size comparable to the line widths, and therefore are hardly visible.

Figure 3 shows the electronic heat capacities in the studied samples. They differ by ~5% at high electronic temperatures, but the relative difference reaches up to 40% at low electronic temperatures *T_e_* < 5000 K. The results suggest that the parameters of bulk gold may be used for simulations of nano-sized samples at high electronic temperatures.

### 3.2. Nonthermal Effects

Gold ultrathin layer expansion and ablation were discussed in [14], which showed emission of the outermost atomic layers at the deposited dose of 2 eV/atom. Inside the remaining layer, destabilization of the lattice and ultrafast atomic disorder (melting) take place.

**Figure 4:**
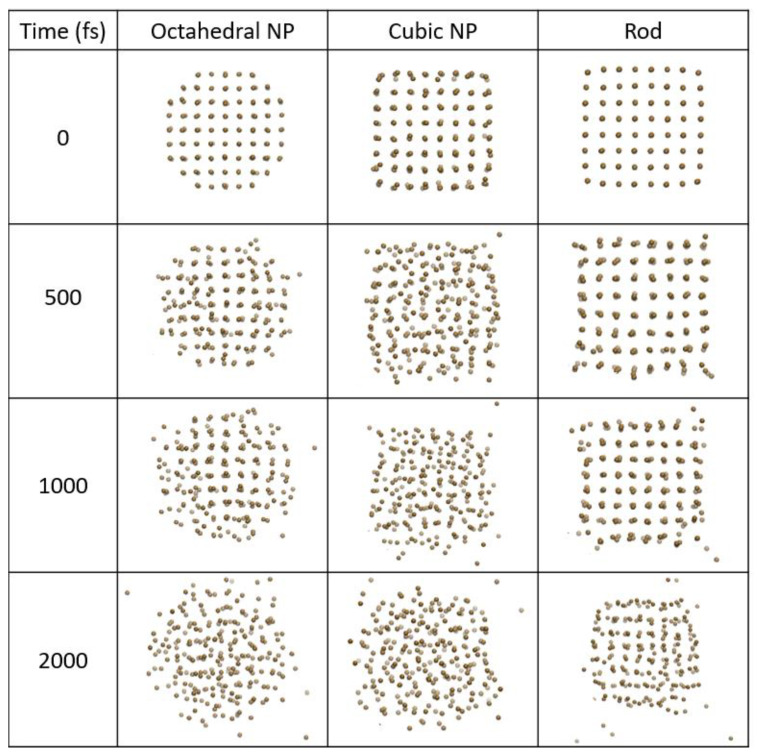
Snapshots of gold nanoparticle (octahedral and cubic, 1.62 nm) and rod (1.6 nm) irradiated with 2 eV/atom dose calculated with XTANT-3.

Here, we demonstrate the dynamics of the process of nonthermal expansion, induced by the increase in the electronic temperature (and hence, pressure), in gold NPs and a nano-rod, see Figure 4. The octahedral and cubic NP reaction, as well as that of a rod, is similar to the nano-layer. The nano-rod emits atoms mainly from the edges, which are most loosely bound. Similarly, a cubic NP emits atoms from the corners and edges. An octahedral NP does not seem to have preferential atoms emission sites.

After the first ablation/emission from the outermost atomic layer, the insides of the nano-samples start to destabilize and disorder. The cubic NP disorders the fastest (within 500 fs) among all studied samples at the same deposited dose of 2 eV/atom. We assume an unpolarized laser pulse, neglecting possible effects of preferential directionality of excitation, which could make a difference for strongly anisotropic nano-objects.

Octahedral NP disorder takes longer (~1 ps), whereas the nano-rod takes the longest time to disorder (~2 ps). Nevertheless, all these times are significantly faster than the electron–phonon equilibration times that take a few tens of picoseconds. It is the nonthermal effects that trigger the atomic disorder, not the thermal atomic heating via electron–phonon coupling.

## 4. Discussion

To reach the electronic temperatures above *T_e_*~15,000–20,000 K in gold, it requires the deposited doses of ~4 eV/atom. At such doses, nonthermal effects may take place within the simulation times that we use to extract coupling parameters, see more detailed discussion in Appendix A. In this case, the extracted coupling parameter no longer corresponds to the room temperature, *T_a_* = 300 K. With the increase in the atomic temperature, the coupling parameter increases nearly linearly [10]. This affects the data at high electronic temperatures (typically, at temperatures above those shown in Figure 2). As a word of caution, we marked the region that might be affected by this effect with the shaded area in Figure 2. In this region, we see some deviation from a nearly linear dependence in the curve for octahedral NP. This deviation is due to the increase in the atomic temperature caused by nonthermal expansion.

Let us also note that at low electronic temperatures (below *T_e_*~1500–2000 K, shown with a dotted line in Figure 2), the results on the coupling parameter in the bulk may be unreliable because only Gamma-point is used for the TB calculation of the electronic energy levels (eigenstates). As discussed in [10], this may be insufficient to accurately sample the band structure within the width of the smearing of the electronic Fermi distribution function at low temperatures. The bulk data at such low electronic temperatures are, therefore, marked with the dotted line.

In nano-sized samples, ultrafast energy deposition increases electronic temperature and, hence, electronic pressure. As was discussed in detail in [14], it is typical for finite-size metals to experience nonthermal expansion and ensuing instabilities. The damage process is a result of electronic pressure that accelerates atoms into expansion. The lattice in an expanded state becomes unstable and may collapse either into a different solid-state or disorder [14]. A similar notion was discussed in earlier works on tungsten [19,20] and corroborated by DFT simulations in [30] accounting for a uniaxial material expansion near a surface. As was also suggested in earlier works, this effect may be incorporated into classical MD simulations using electronic pressure (or so-called electron blast force) [31,32,33], or effective electronic pressure terms in a two-temperature model [34].

The simulations in [14] were performed within the Parrinello–Rahman MD simulations (NPH ensemble). The results presented in Section 3.2, Figure 4, are free from adjustable parameters (such as Parrinello–Rahman supercell effective mass) and demonstrate the effect of nonthermal effects directly. The current work validates the methodology and results of [14] and demonstrates the timescales of such nano-object expansion that may be directly validated in future experiments.

**Figure 5:**
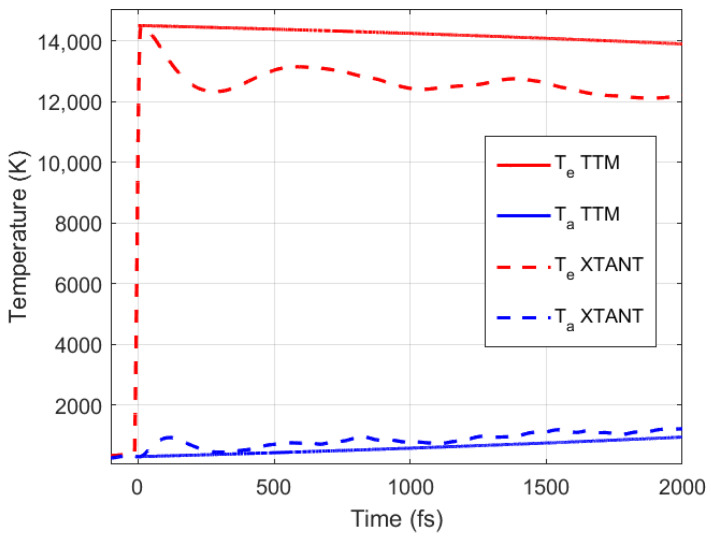
Electronic and atomic temperatures in octahedral nanoparticle irradiated with 2 eV/atom dose calculated with XTANT-3 compared to those calculated with the TTM.

Finally, let us remark on the interplay of the thermal and nonthermal effects in an NP response to irradiation. For illustration, we compare a full XTANT simulation with a simple two-temperature model (TTM) simulation [8], see Figure 5. Instantaneous uniform heating of the electronic system was assumed and described within the TTM formalism as

$$\{\begin{array}{c} C_{e}\left(T_{e}\right)\frac{\partial T_{e}}{\partial t}=-G\left(T_{e},T_{a}\right)\left(T_{e}-T_{a}\right)\\ C_{a}\frac{\partial T_{a}}{\partial t}=G\left(T_{e},T_{a}\right)\left(T_{e}-T_{a}\right), \end{array}$$

with the initial conditions *T_e_* = 14,500 K and *T_a_* = 300 K. Here, we assumed a constant atomic heat capacity $C_{a}$ and independence of the electronic heat capacity $C_{e}$ of the atomic temperature [10].

Although the TTM calculations use the coupling parameter *G*(*T_e_*, *T_a_*) and electronic heat capacity reported in this work (dependence on the atomic temperature in gold was reported in [10])—i.e., identical to those in XTANT-3 simulation—the results are noticeably different.

We can see that, as expected, the TTM does not reproduce the large oscillations seen in XTANT-3 that are the results of nonthermal expansion, triggered by electronic pressure increase. This effect leads to atomic acceleration that affects the temperature (and, hence, the coupling parameter). Such a nonlinear synergy of thermal and nonthermal effects leads to a faster drop of the electronic temperature and a correspondingly faster increase in the atomic one. A similar effect in bulk insulators was recently reported in [35], where a nonthermal band gap collapse was triggering atomic acceleration and an interplay with the thermal electron–ion coupling.

## 5. Conclusions

Calculations with nonadiabatic tight-binding molecular dynamics (XTANT-3 code) predicted that the electron–phonon coupling in gold does not depend on the sample size, dimensionality, and geometry at electronic temperatures above *T_e_*~7000 K. Gold bulk, nano-layer, rod, and two kinds of nanoparticles studied (octahedral and cubic) all showed nearly identical coupling parameters. At lower electronic temperatures, *T_e_* < 7000 K, a minor difference in sample coupling parameter is obtained. The electronic heat capacities are very close among the modeled samples at high electronic temperatures, and differ by some 40% at *T_e_* < 5000 K. These results suggest that the parameters of bulk gold may be used for simulations of nano-sized samples at high electronic temperatures.

We also demonstrated that nonthermal effects are important in gold nano-sized samples: an ultrafast increase in the electronic temperature leads to an increase in the electronic pressure, which induces expansion and atomic acceleration (hence, an increase in the atomic temperature). This expansion and corresponding atomic heating for nano-sized samples take place within a few hundred femtoseconds—much faster than the electron–phonon coupling. The nonthermal expansion leads to material ablation and ultrafast destabilization and disordering inside all nano-gold samples studied.

A standard two-temperature model (TTM), using the calculated electron–phonon coupling parameter and electronic heat capacity, is unable to reproduce the atomic heating timescales in nanoparticles. The TTM fails in this case because the dominant effect leading to atomic acceleration is the nonthermal expansion and not the electron–phonon coupling. We thus conclude that the effects of the electronic pressure must necessarily be taken into account in modeling metallic nano-objects under ultrafast irradiation.

## Figures and Tables

**Figure A1:**
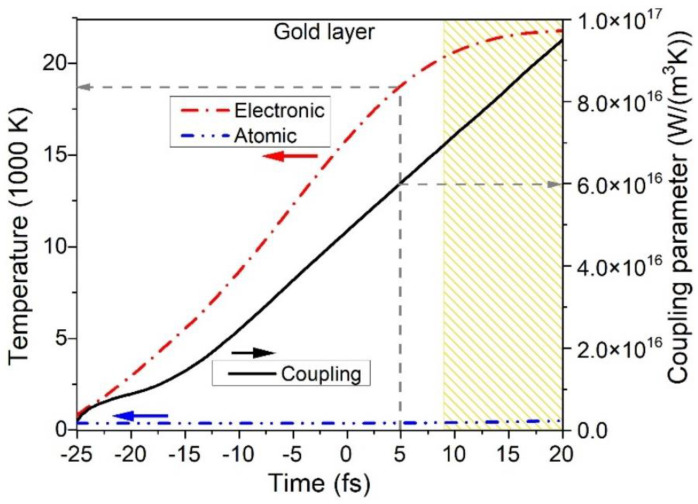
An example of electronic and atomic temperatures, and coupling parameter evolution, calculated with XTANT-3 in a gold nanolayer. Grey arrows point to the values of both quantities at the same time instant. Shaded area indicates the region with noticeable atomic heating due to nonthermal effects taking place.

**Figure A2:**
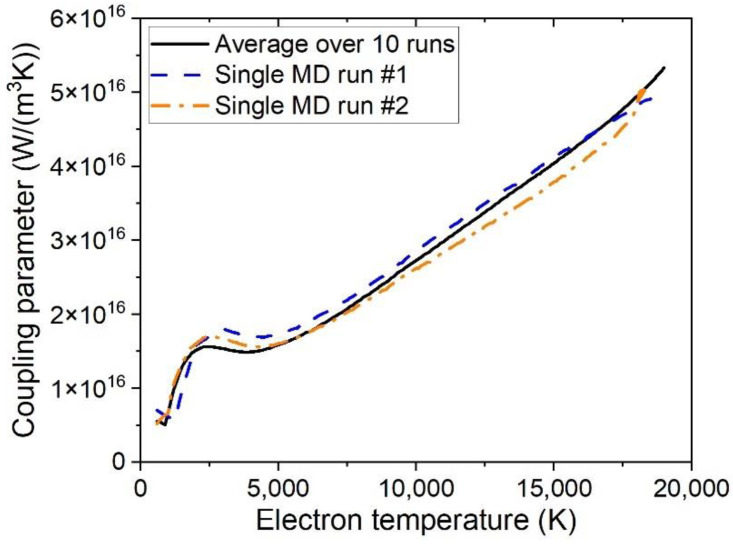
An example of the electron–phonon coupling parameter calculated in single MD simulation runs, compared with the one averaged over 10 runs.

## Data Availability

The data produced in this study are available from the authors upon reasonable request.

## Appendix A. Methodology of Electron–Phonon Coupling Calculation

The developed methodology uses tight-binding molecular dynamics simulation to calculate the electron–phonon coupling parameter. Note that the electron transition rates *w_ij_* (Equation (3)) are non-local in time; even though we used the overlap matrix, $S_{\alpha ,\beta }$, on the same timestep, the wave functions, $\psi_{i}$, from two consecutive time steps are used unavoidably. Thus, they require an MD simulation run to have temporal evolution of the wave functions.

At each consecutive time step of the simulation, we calculate the electron transition rates and correspondingly the coupling parameter. To extract the coupling parameter as a function of the electronic temperature, we smoothly increase the electronic temperature in the simulation within a few tens of femtoseconds duration. Then, having the data for the electron temperature (*T_e_*(*t*)) and coupling parameter (*G*(*t*)) as functions of time within the same simulation run, we can find the correspondence between the two and construct the function *G*(*T_e_*), see an example in Figure A1. The electron heat capacity *C_e_*(*T_e_*) is extracted in the same way, with the difference being that it is local in time.

To exclude the nonthermal effects, the electron temperature increase should take place within the timescales shorter than the nonthermal atomic heating and expansion of a nano-object. This limits the electron temperature increase to the times of about 10–20 fs (FWHM). For longer increase times, nonthermal effects such as atomic acceleration become non-negligible, which affects the calculated coupling parameter, since it is a function of the atomic temperature, density, and structure [10]. Note that even with the increase in the electronic temperature with 20 fs FWHM, shown in Figure A1, there is a slight increase in the atomic temperature at the latest times of ~15–20 fs. That shows that at the highest electronic temperatures (reached at the same time) there is some contribution of the nonthermal effects, as mentioned in the Discussion section.

The electron–ion coupling parameter is rather sensitive to the particular state of the system, and thus requires an averaging over a few simulations. To exclude oscillations of the defined macroscopic quantities associated with the finite-size effects in the simulation box, and an artificial influence of a particular state of the atomic ensemble, we perform 10 simulation runs. Each run starts with different initial velocities of the atoms, sampled randomly following the Maxwellian distribution. We also chose different durations of the electronic temperature increase in the interval between 10 to 20 fs, and the aimed electronic temperature between 20,000 and 25,000 K. An example of a comparison of the coupling parameter in a single MD simulation run vs. the averaged one is demonstrated in Figure A2. We see that the averaging makes a noticeable difference.
